# Supplementary material for: Adherence to COVID-19 preventive measures and associated factors in Oromia regional state of Ethiopia
Source: PLoS One. 2021 Oct 20;16(10):e0257373. doi: 10.1371/journal.pone.0257373 (PMC8528333; doi:10.1371/journal.pone.0257373)
Supplement: S1 Questionnaire — (DOCX) [file pone.0257373.s002.docx]

**Annexes**

**Questionnaire to assess Magnitudes of Compliances to Facility Based Maternal and Child Health Care Services During COVID-19 Pandemic and Associated Factors among Selected Hot Spot Zones of Oromia Regional State**

Hello. My name is ____________and I am here on behalf of the Oromia Region Health Bureau. I am here to collect information about general Socio-demographic characteristics; Knowledge related to COVID-19, Attitude questions about COVID-19, Adherence to utilizations of facility based MCH service utilization and its associated factors of the study participants. Specifically, about Factors influencing adherence to COVID-19 preventive measures and utilization of facility based health care services and utilizations of essential health care services among randomly selected Community‘s in the Oromia Regional State Administration, and requesting you to participate in this study, which would require your response to an interview with some, related issues.

**Title of the Study:** Magnitudes of Compliances to Facility Based Maternal and Child Health Care Services During COVID-19 Pandemic and Associated Factors among Selected Hot Spot Zones of Oromia Regional State

**Background:** Scholars suggest that the COVID-19 disease was first identified during the outbreak of severe acute respiratory syndrome in Wuhan, China, in December 2019. Subsequently, it becomes a serious infectious disease, caused by the Severe acute respiratory syndrome coronavirus 2 (SARS-Co V-2) and declared as a pandemic (public health emergency of international concern) by the World Health Organization (WHO) on March 10, 2020, which was the third since the establishment of the WHO, following the Hong Kong flu in 1968 and H1N1 flu in 20091. In Africa, the first confirmed case of COVID-19 was reported in Egypt on February 14, 2020.

**Benefit**: There is no any direct benefit given to the study participants for this study. However, the study findings would be used to design and implement good organizational culture in public sector employees’ in the future.

**Risk**: There are no risks posed to the participants of the study

**Method of selection**: You are selected for this study randomly/by chance.

**Confidentiality:** Their name was not written and never used in connection with any information you give to me. All information given by you will be kept strictly confidential.

**Rights:** Your participation is purely voluntary and you are not obligated to answer any question you do not wish to answer. If you feel discomfort with the interview, you can withdraw any time after you get involved in the study.

**Duration of interview**: This interview will take about 20 minutes.

Could I have your Permission to continue?

If yes, continue the interview.

If no, skip to the next participant by writing the reasons for his/her refusal.

For any questions you have, you can contact principal Investigator Dr Sileshi Garoma 0911076012

Interviewer: Code____________Name_______________________signature_____________ Date of interview _____________Time started ________________Time completed_________ Result of interview: 1. Completed 2. Respondent not available 3. Refused 4. Partially completed Checked by: Supervisor: Name ____________________________ Signature____________

Date _____________________

|  | **Section 1: Socio-demographic characteristics** | | | | | | | | | |  | |
| --- | --- | --- | --- | --- | --- | --- | --- | --- | --- | --- | --- | --- |
| No. | QUESTIONS | | RESPONSE CATEGORY | | | | | | | | SKIP | |
| q 101 | Cluster | | 1. Agrarian  2. Agro-Pastoralists  3. Pastoralist | | | | | | | |  | |
| q 102 | Residence or Location: | | 1. Urban/Town  2. Rural/Woreda | | | | | | | |  | |
| q 103 | Sex of the respondents | | 1. Male  2. Female | | | | | | | |  | |
| q 104 | Marital Status | | 1. Single  2. Married  3. Widowed/Divorced  4. Separated | | | | | | | |  | |
| q 105 | Age of respondents: | | ______ Yrs | | | | | | | |  | |
| q 106 | Religion | | Orthodox  Muslim  Protestant  Catholic  Others/specify__________ | | | | | | | |  | |
| q107 | Ethnicity | | 1. Oromo 2. Amhara 3. Tigre 4. Others/Specify__________ | | | | | | | |  | |
| q 108 | Occupation of respondents | | 1. Farmer or pasturalist  2. Merchant  3. Student  4. Gov./NGO worker  5. Others specify_________ | | | |  | | | | | |
| q 109 | Level of education | | 1. Illiterate  2. Read and write  3. Primary  4. Secondary  5. Colleges and above  6. Others/specify________ | | | |  | | | | | |
| q 110 | Estimated annual income | | ______ ETB | | | |  | | | | | |
| Section 2: Knowledge related to COVID-19 | | | | | | | | | | | | |
| q 201 | Have you ever heard about the new COVID-19 disease? | | | | | | | | | 0= No  1= Yes | | q401 |
| q202 | Do you believe in the existence of COVID-19 in your area? | | | | | | | | | 0= No  1= Yes | |  |
| q 203 | How dangerous do you think the new COVID-19 is? | | | | | 0. Not dangerous 1. Dangerous 2. Very dangerous 3. Killers 4. Others/specify______ | | | | | |  |
| q 204 | Source of information about COVID-19 | | | | 1. Radio 2. TV 3. Other Social Media 4. Health unit/Health care worker 5. Family members 6. Friends 7. Others/Specify ____ | | | | | | |  |
| q205 | What kind of information have you received about the COVID-19? Multiple answer possible) | | | | 1. Protection steps 2. Symptoms 3. Transmission 4. Self-care 5. Risks/complications 6. Others/specify_____ | | | | | | |  |
| q 206 | I am confident about the information disseminated by the Ethiopian Ministry of Health or Oromia Health Bureau about COVID-19 | | | | | | | | | 0. No  1. Yes | |  |
| q 207 | Do you think that it is important to take actions to prevent the spread of COVID-19 in your community? | | | | | | | | | 0. No  1. Yes | |  |
| q208 | Do you know how to prevent becoming sick from the new COVID-19? | 1. Wash your hands regularly using hand rub or soap and water 2. Avoid hand shaking and hugging 3. Cover your mouth and nose when coughing or sneezing 4. Avoid close contact with anyone who has a fever and cough 5. Cook all animal products 6. Avoid unprotected direct contact with live animals and  Surfaces in contact with animals 7. Don't know 8. Other/Specify__________ | | | | | | | | | |  |
| q209 | What kind of measures has you or your family taken to prevent COVID-19 in the recent days? | 1. Wash your hands regularly using hand rub or soap and water 2. Avoid hand shaking and hugging 3. Cover your mouth and nose when coughing or sneezing 4. Avoid close contact with anyone who has a fever and cough 5. Cook all animal products 6. Avoid unprotected direct contact with live animals and  Surfaces in contact with animals 7. Don't know 8. Other/Specify__________ | | | | | | | | | |  |
| q210 | What would you do if you or someone from your family gets sick with the new COVID-19? | 1. I will look for a more experienced relative to advise me on what to do 2. I would go to the hospital / health unit 3. I would go to the neighborhood nurse 4. I would go to buy medicines at the market /Pharmacy 5. I'm going to look for the traditional healer 6. I would stay in quarantine 7. Other/specify _____________ | | | | | | | | | |  |
| q 211 | Do you think you are at risk of getting sick with the new COVID-19? | | | | | | | | 0= No  1= Yes | | |  |
| q 212 | Do you know how the COVID-19 is spread? | | | 1. Blood transfusion 2. Droplets from infected people 3. Airborne 4. Direct contact with infected people. 5. Touching contaminated objects/surfaces  6. Sexual intercourse contact  7. Contact with contaminated animals 8. Mosquito bites  9. Eating contaminated food 10. Drinking unclean water 11. Don't know | | | | | | | |  |
| q 213 | What are the main symptoms? | | | 1. Fever  2. Cough  3. Shortness of breath and breathing difficulties 4. Muscle pain  5. Headache  6. Diarrhea 7. Don't know 8. No symptoms 9. Other:_____________ | | | | | | | |  |
| q214 | Covering the nose and mouth while coughing | | | | | | | 0= No  1= Yes | | | |  |
| q 215 | Avoiding crowds in public places | | | | | | | 0. No  1. Yes | | | |  |
| q 216 | Frequent cleaning and disinfecting surfaces | | | | | | | 0.No  1. Yes | | | |  |
| q 217 | Keep at least two meter distance between people | | | | | | | 0. No  1. Yes | | | |  |
| q 218 | Avoid direct contact with colleagues (others) | | | | | | | 0. No  1. Yes | | | |  |
| q 219 | If most of the above answers are no, did you practice any of them since COVID-19 occur | | | | | | | 0. No  1. Yes | | | |  |
| q 220 | If No, what might be the possible reason | | | | | | | Specify _____ | | | |  |
| **Section 3: Attitude related questions about COVID-19** | | | | | | | | | | | | |

|  |  | Agree | Neutral | Disagree |
| --- | --- | --- | --- | --- |
| q 301 | COVID-19 is a killer disease |  |  |  |
| q 302 | COVID-19 is preventable |  |  |  |
| q 303 | The government is responsible for implementing the preventive measures and utilization of facility based health care services |  |  |  |
| q 304 | The community is responsible for preventive COVID-19 |  |  |  |
| q 305 | Individual are responsible to apply all the preventable to apply all the preventive measures and utilization of facility based health care services told by FMOH/RHB /health care |  |  |  |

| **Section 4:** Individual compliance status to facility based Reproductive, Maternal, Neonatal and Child health care (RMNCH) services during COVID-19 Pandemic | | | | | | |
| --- | --- | --- | --- | --- | --- | --- |
| q 401 | I can visit health care facilities whenever feeling a sense of pain or discomfort | | | | 0. No  1. Yes |  |
| q 402 | Do you or your family member have experienced any facility based Reproductive, Maternal, Neonatal and Child health care (RMNCH) services like family planning, antenatal care or postnatal care | | | | 0. No  1. Yes | q403 |
| q 403 | If yes to q 402, what did you do at that time? | 1. Visit health care 2. Find traditional healer or home care 3. Find religious person or pray 4. Do nothing | | | |  |
| q 404 | If no to q 402, what was your reason? | 1. Fear of COVID-19 2. Lack of access of services 3. Fear of stigma 4. Spiritual believer 5. Others/Specify_______ | | | |  |
| q 405 | Before the COVID-19 when you or your family member get sick what did you do? | 1. Visit health care 2. Find traditional healer or home care 3. Find religious person or pray 4. Do nothing | | | |  |
| q406 | If q 405 is out of visit health care ask why? | 1. Fear of COVID-19 2. Lack of access of services 3. Fear of stigma 4. Spiritual believer 5. Others/Specify_______ | | | |  |
| q407 | If q405 is visit health care, do you think that you will get good care? | | | 0. No  1. Yes | |  |
| q408 | Is there anyone in your family who needs health care (ANC, immunization, and other health services) from the last 6 months to know? | | | 0. No  1. Yes | |  |
| q409 | If Yes to q408, ask are they visiting health care | | | 0. No  1. Yes | |  |
| q410 | If No to q408, ask why? | | 1. Fear of COVID-19 2. Lack of access of services 3. Fear of stigma 4. Spiritual believer 5. Others/Specify_______ | | |  |
