# Supplementary material for: Adherence to COVID-19 preventive measures and associated factors in Oromia regional state of Ethiopia
Source: PLoS One. 2021 Oct 20;16(10):e0257373. doi: 10.1371/journal.pone.0257373 (PMC8528333; doi:10.1371/journal.pone.0257373)
Supplement: Attachment — Submitted filename: Reviewer_Bob.docx [file pone.0257373.s004.docx]

**Methods**

It is well written and systematic. Readers can easily understand.

- Sample size determination of the quantitative data is scientifically drawn. However, cite/ reference the formula.

**Results**

Results are captivating. However, improvement can be made as follows:

- The estimated annual income of the respondents can be also be put in US dollars to give and an easier understanding to the international community
- What is the difference between rural and urban communities with regard to the knowledge of COVID-19 including its preventive measures. Can author show proportions for rural and urban differently?
- When pointing out results for factors associated with adherence, the authors state an explanation of how the results were got (methods). I would suggest that that part removed.
- Check out this statement “Accordingly, compared to study participants who attended Colleges and above, being Illiterate [AOR, 38; 95% CI: 0.15, 0.93]”. Here you need to correct that AOR. It seems incorrect.
- Include AOR value in this statement “Being merchant were less (AOR; 95% CI: 0.29, 0.96]”
- Include percentage sign in this statement “…above knowledge related variables and accordingly 1606 (58.4) have good level of knowledge”

**Discussions**

Well structured, however, make improvements in the following:

- Cite this stamen “This may be because the developing countries use social media less than developed countries and minimize disruption caused by the corona virus”
- What is the implication of the knowledge level of 58%
- Please share why you think this study had lower favorable attitudes towards COVID-19 Preventive measures as compared to other studies, including that in Ethiopia. In addition, show the implication of this finding.
